# Assessing public interest in sunscreen safety during pregnancy and lactation

**DOI:** 10.1097/JW9.0000000000000018

**Published:** 2022-05-19

**Authors:** Sarah Shareef, Wyatt Boothby-Shoemaker, Alyssa Abdelnour, Evien Albazi, Kurt Ashack

**Affiliations:** Michigan State University College of Human Medicine, East Lansing, Michigan; Michigan State University College of Human Medicine, East Lansing, Michigan, Dermatology Associates of West Michigan, Grand Rapids, Michigan

**Keywords:** chemical, mineral, physical, pregnancy issues, sunscreen

**What is known about this subject regarding women and their families?**

- Sunscreen is widely used to reduce the risk of skin cancer. In May 2019, an original investigation by Matta et al. reported active ingredients of chemical sunscreens found systemically in greater concentrations than those recommended by the Food and Drug Administration (FDA). Moreover, with recent recalls of sunscreen contaminated with benzene, there has been increased public concern regarding sunscreen safety.
- The American Academy of Dermatology (AAD) does not have recommendations for sunscreen specific to pregnant women or those who are nursing.

**What is new from this article as messages for women and their families?**

- There is an increased interest based on online searches about sunscreen safety in pregnancy and nursing evident through Google Trends analysis from May 2017 to April 2019 compared with May 2019 to April 2021.
- Although easily accessible, discussion on social media regarding sunscreen use in pregnancy demonstrated heterogeneity in the recommendations and does not exclusively include information from board-certified dermatologists, thus guidance may not be from reliable sources.
- Given increased search interest in sunscreen safety through social media, this may be an area where recommendations from expert, dermatologic bodies such as the AAD may provide direction for clinicians caring for pregnant and breastfeeding individuals.

## Dear Editors,

There has been an increased concern regarding sunscreen’s long-term effects due to the systemic absorptive properties of certain chemical ingredients. In 2019, a preliminary study found plasma concentrations of chemical byproducts above the Food and Drug Administration (FDA) recommended limit. This study’s findings sparked interest in comparing chemical sunscreens to those utilizing physical mineral blockers.^1^ Currently, there are no known studies evaluating the interest on sunscreen selection among pregnant and/or nursing individuals. Thus, we completed an internet-based analysis to assess for changes in sunscreen recommendations for this demographic before and after May 2019.

Google Trends can be used to compare Google search queries.^2^ We used this source to obtain United States search volumes between the dates of April 30, 2017–May 1, 2019, and May 1, 2019–April 30, 2021, while using the search terms: “sunscreen for pregnancy,” “safe sunscreen pregnancy,” “sunscreen pregnancy,” “sunscreen lactation,” “safe sunscreen breastfeeding,” and “sunscreen nursing.” A student’s t-test was performed on results with a high enough search yield on Google and YouTube between the specified time intervals. The first 50 results were analyzed for sunscreen recommendation type and whether recommendations came from a dermatologist or nondermatologist. Advertisements, duplicates, and websites without publication dates were excluded from analysis.

We found a statistically significant increase (*P* < .01) in Google searches for “sunscreen for pregnancy,” “safe sunscreen pregnancy,” and “sunscreen pregnancy” between 2019–2021 compared with 2017–2019. Search terms for sunscreen use in breastfeeding, lactation, and nursing did not consistently return a measurable search volume yield from Google Trends. alone. Following search and exclusion criteria for “safe sunscreen pregnancy” and “safe sunscreen lactation,” there were 65 YouTube videos evaluated and 45 webpages from Google queries. For Google searches, a higher proportion of recommendations was found for mineral sunscreens after May 2019 (86%) compared with dates prior (60%). Moreover, there was a smaller proportion of recommendations for both chemical and mineral sunscreens after May 2019 (11%) in contrast to dates prior to May 2019 (30%) (Table 1). YouTube analysis indicated recommendations for mineral sunscreen alone in 71% of videos after May 2019 in contrast to 57% before May 2019. However, total proportion of chemical sunscreen recommendations increased from 0% to 10% after May 2019 (Table 1).

**Table 1.** Recommendations from Google articles and YouTube videos from searches for “safe sunscreen pregnancy” and “safe sunscreen lactation”

Source	Time frame	Nondermatologist recommendations, n (%)	Dermatologist recommendations, n (%)	Total	Mineral sunscreen, n (%)	Chemical sunscreen, n (%)	Both, n (%)	Sunscreen endorsement, but no specific recommendation, n (%)	Total
YouTube	Before May 2019	3 (43)	4 (57)	7 (100)	4 (57)	0 (0)	3 (43)	0 (0)	7 (100)
	After May 2019	28 (48)	30 (52)	58 (100)	41 (71)	6 (10)	11 (19)	0 (0)	58 (100)
Google	Before May 2019	10 (100)	0 (0)	10 (100)	6 (60)	0 (0)	1 (10)	3 (30)	10 (100)
	After May 2019	24 (69)	11 (31)	35 (100)	30 (86)	0 (0)	1 (3)	4 (11)	35 (100)

Included are the number of recommendations provided and the type of sunscreen recommended (mineral, chemical, neither, or no specific recommendation besides sunscreen) by both nondermatologists and dermatologists. Duplicated articles or sources with no date of publication were excluded from the analysis.

In summary, the increase in recommendations for use of mineral sunscreens after May 2019 coincides chronologically with reports of increased systemic absorption of chemical sunscreens in individuals. These findings coincide chronologically with reports of increased systemic absorption of chemical sunscreens in individuals. This has prompted the FDA to recommend additional research on organic ultraviolet filters to support further policy recommendations about sunscreen to support the health of this population.^1^ Public interest in sunscreen safety is increasing and it would be helpful if the American Academy of Dermatology (AAD) provided guidance regarding which sunscreens are safe for use for pregnant and lactating women. An analysis of Google trends reveals that sunscreen recommendations are widely varied and not necessarily based on expert opinion. Limitations to this study include a small sample size, rapid addition of content to social media platforms, and the possibility of content not representing overall patient attitudes.

## Conflicts of interest

None.

## Funding

None.

## Study approval

N/A
